# Editorial: Crosstalk between innate and adaptive immunity in colorectal cancer: Implications for immunotherapy

**DOI:** 10.3389/fimmu.2022.1129001

**Published:** 2023-01-11

**Authors:** Anne Jarry, Nadine Gervois, Celine Bossard, Markus Germann

**Affiliations:** ^1^ Nantes Université, Univ Angers, INSERM, CNRS, Immunology and New Concepts in ImmunoTherapy, INCIT, UMR 1302/EMR6001, Nantes, France; ^2^ LabEx IGO, Université de Nantes, Nantes, France; ^3^ Department of Biomedicine, University Hospital Basel, Basel, Switzerland

**Keywords:** colorectal cancer (CRC), innate immunity, adaptive immunity, immunotherapy, microbiota, anti-tumor response

Colorectal cancer (CRC), one of the most frequent solid malignancies, is a heterogeneous disease, influenced by complex interactions between tumor cells, immune cells of the tumor microenvironment (TME) and the gut microbiota. These interactions lead to the development of a local immune response that can have immunosuppressive or immunostimulatory properties. Although cytotoxic CD8 T cells are undoubtedly considered to be the most potent effectors of the anti-cancer immune response, their efficacy depends on a large number of parameters, including the diversity and frequency of immune and non-immune cells present within the TME as well as their spatial organization, without omitting the influence of microbial dysbiosis. In CRC, albeit a high CD8 T cell infiltrate correlates with a favorable outcome and with response to immunotherapy using immune checkpoint inhibitors (ICi), only a minor subset of CRC patients is responsive to ICi. A better understanding of the complex interplay between the innate and adaptive arm of the immune system that may influence anti-tumor responses could lead to improve immunotherapy efficacy.

In this research topic of Frontiers in Immunology, we have collected reviews and original works that provide an overview as well as new insights on the interactions between tumor cells, innate and adaptive immune cells and the microbiota.

## Interaction between innate and adaptive immune cells within the tumor microenvironment of colorectal cancer

Ghilas et al. provide a comprehensive overview and update of the complex interplay between macrophages, dendritic cells, innate lymphoid cells and epithelial cells in intestinal immune homeostasis as well as in the context of chronic intestinal inflammation and colon cancer. The authors show how aberrant activation of both innate and adaptive arms of the immune system can promote chronic inflammation, an immunosuppressive environment and increase the risk of colon cancer development. In CRC, the various subsets of tumor-associated macrophages (TAM) within the TME are discussed, with emphasis on the plasticity of TAM, on the diverse phenotypic/functional states that may exist between the classical M1 and M2 “endotypes” and on the conflicting reports regarding the correlation of TAM infiltration with patient survival prognosis. The authors also mention the important role of tumor-associated dendritic cells (DC) which, although representing only a minority of myeloid cells in the TME, participate in priming adaptive immune responses. This leads, depending on the DC subset involved (classical or plasmacytoid DC) and their response features, either to tumor progression or regression. Finally, the authors highlight recent advances and discoveries in the evolving field of innate lymphoid cells (ILCs) in CRC. Indeed, ILCs are key intermediaries during intestinal homeostasis, intestinal inflammation and colon cancer. In colon cancer, ILCs act as a double-edged sword, able to promote or inhibit tumorigenesis and have a different impact on disease outcome according to the subset involved and its cytokine production. Overall, a deeper understanding of the contribution of innate immune cell subsets and their interactions with epithelial cells and the microbiota could offer new therapeutic strategies, including combination therapies, for the treatment of inflammatory bowel diseases and colon cancer.

In CRC, the variation of TME composition plays a critical role in cancer development and affects the efficacy of immunotherapy. Within the TME, ectopic lymph nodes, so-called tertiary lymphoid structures (TLS) as they share some structural and functional features with secondary lymphoid organs, are present in epithelial-derived cancers. It has been reported that these TLS have an impact on patient prognosis, may be important for anti-tumor immunity, and can predict response to immune checkpoint inhibitors. In CRC, the prognostic value and role of TLS is still controversial. Wang et al. examined in two independent cohorts of CRC patients the prognostic impact of peritumoral TLS density and tumor stroma percentage (TSP) in non-metastatic colorectal cancer. The authors assessed the phenotype of cells present in TLS and showed that high TLS density and low TSP were independent and favorable prognostic factors in non-metastatic CRC patients. The authors also established a Nomogram including the TLS, TSP and TNM stage that could guide clinicians to identify non-metastatic CRC with a poor prognosis and predict the probability of recurrence free survival within 2-5 years in these patients.

## Interaction between gut microbiota and tumor cells in colorectal cancer

Xing et al. provide a comprehensive review focusing on the complex interactions between gut microbiota and cancer cells in CRC *via* interactions between innate and adaptive immune signaling pathways including innate immune cells and NFkB, IFN type I and inflammasome pathways. An overview of cancer-promoting and cancer-inhibiting microbiota species able to directly interact with host immunity is provided, as well as metabolic products of the microbiota that can indirectly trigger specific immune responses. An overall profiling of gut microbiota and metabolites could serve as diagnostic markers to assess CRC risk in healthy individuals and prognosis in CRC patients. The authors also discuss the development of microbiota-based therapies such as fecal transplantation, specific elimination of detrimental species, or targeted bacteriophage therapy, that can be used to improve the efficacy of immunotherapy in CRC.

Flickinger et al. address the challenging issue of bacteria-based vaccine immunotherapy to boost immune responses in cancer. The authors examined in a mouse model the immunogenicity of a *Listeria monocytogenes* (Lm) vaccine against the CRC tumor antigen guanylyl cyclase C (GUCY2). Surprisingly, they show for the first time that Lm-derived peptides compete with the targeted tumor antigen presentation and immunogenicity, limiting the CD8 T cell response towards the dominant GUCY2C epitope and thus the effectiveness of this Lm-based vaccine. In addition, they show that the use of a peptide modified by substitution of an anchor residue enhancing peptide-MHC stability can rescue a robust anti-tumor CD8 T cell response. This study unveils mechanisms that limit the efficacy of Lm-based vaccines and suggests novel approaches such as immunogenic peptide analogue or prime-boost, to maximize the efficacy of Lm-based vaccines.

We thank all authors of this Research Topic, as well as the reviewers for their contributions.

## Author contributions

AJ and NG wrote the editorial. All authors approved the submitted version.

